# A Scoping Review Protocol of Lung Ultrasound in Pediatric COVID‐19: Clinical Utility and Lessons for Future Pandemics

**DOI:** 10.1002/hsr2.72310

**Published:** 2026-04-14

**Authors:** Yusuf Syaeful Nawawi, Anistyaning Wahyu Adhie

**Affiliations:** ^1^ JBI, School of Public Health, College of Health Adelaide University Adelaide Australia; ^2^ Department of Radiology Dr. Moewardi Hospital Surakarta Indonesia

**Keywords:** COVID‐19, lung ultrasound, pediatric, scoping review

## Abstract

**Background and Aims:**

Lung ultrasound (LUS) is an ultrasound‐based procedure used to evaluate thoracic structures, including the pleura and lung parenchyma. It has become an increasingly important tool for diagnosing and monitoring respiratory diseases, including in pediatric populations. The recent COVID‐19 pandemic had created unprecedented challenges in respiratory diagnosis and care, accelerating the use of LUS as a rapid, practical, and safe examination method, particularly suitable for children. This scoping review aims to map and synthesize evidence on the use of LUS in pediatric COVID‐19, identify research gaps, and inform future studies.

**Methods:**

This review will be conducted in accordance with JBI methodological guidance for scoping reviews and reported by following the preferred reporting items for systematic reviews and meta‐analyses extension for scoping reviews (PRISMA‐ScR) checklist. Our strategy will search on major databases, including MEDLINE, Embase, Web of Science, Scopus, and CINAHL, and gray literatures resources. Assessment of the studies will be performed by two independent reviewers against the inclusion criteria. Eligible studies will include pediatric populations (< 18 years) with suspected, confirmed, or recovered COVID‐19, assessing LUS for respiratory evaluation. Studies of any design, language, and methodological quality published from December 2019 onward will be considered. Data will be extracted and synthesized in line with scoping review methodology.

**Results:**

Evidence will be synthesized using a primarily deductive approach with inductive flexibility to identify emerging themes, including clinical applications of LUS, protocols of imaging acquisition and interpretation, and study designs. Findings will be mapped and presented in tables and summarized narratively.

**Conclusion:**

This review will provide a comprehensive overview of LUS use in pediatric COVID‐19, supporting its integration into clinical practice and informing preparedness for future respiratory outbreaks.

**Scoping Review Registration:**

This protocol was registered in Open Science Framework on July 30, 2025 (https://doi.org/10.17605/OSF.IO/XP628).

## 1 Background

Coronavirus disease 2019 (COVID‐19) is caused by the novel severe acute respiratory syndrome coronavirus 2 (SARS‐CoV‐2), an encapsulated single‐stranded RNA virus belonging to the beta coronavirus cluster [1]. COVID‐19 manifests with a wide clinical spectrum, ranging from asymptomatic infection to severe multisystem disease, most commonly affecting the respiratory system [2]. As of mid‐July 2025, more than 778 million cases and over 7 million deaths have been reported worldwide [3]. The World Health Organization ended the COVID‐19 public health emergency on May 5, 2023 [4], yet SARS‐CoV‐2 continues to circulate in various regions in the world with a substantially lower rate [3].

COVID‐19 has affected all age groups, though adults remain the most infected. Children and adolescents are present with less severe illness and fewer deaths compared to adults [5]. However, COVID‐19 infection presents a unique challenge in pediatric care, particularly regarding the safe and accurate assessment of respiratory complications. Radiological diagnosis of COVID‐19 in the pediatric population is challenging. The American College of Radiology recommends using chest X‐ray (CXR) with caution, considering the possible side effects of ionizing radiation, in addition to nonspecific findings of CXR in pediatric COVID‐19. Chest CT is only used for inpatient and symptomatic patients with specific clinical indications and should not be used for the first‐line screening of patients with COVID‐19, especially in the pediatric population. Viral testing by reverse transcriptase‐polymerase chain reaction (RT‐PCR) remains the specific method of diagnosing COVID‐19 as an initial diagnostic test [6].

Chest CT, especially high‐resolution CT, offers superior detail over other imaging modalities to assess the anatomy and morphology of pulmonary structures. Thus, it is considered the gold standard of modalities of chest imaging to examine lung pathologies [7, 8]. However, there have been considerable controversies on the role of chest CT in regard to COVID‐19. Chest CT was recommended as the first‐line diagnostic modality of COVID‐19, especially after a study in China with a large cohort of patients demonstrated higher sensitivity compared to RT‐PCR [9]. However, multidisciplinary expert panels are recommended otherwise. Chest CT should only be reserved for moderate to severe cases of COVID‐19 pneumonia, with certain comorbidities or clinical symptoms favoring disease progression. The use of chest CT for diagnostic purposes of COVID‐19 per se, particularly to replace RT‐PCR, is discouraged [10].

Lung ultrasound (LUS) has been introduced as an alternative imaging modality to examine chest structure. In clinical settings, LUS would particularly be performed through a point‐of‐care approach where urgent clinical diagnosis can be established by performing ultrasound at the bedside, such as in emergency or intensive care settings, to guide prompt management. LUS is a rapid and relatively safe examination, as it uses no ionizing radiation, and it allows for dynamic evaluation and monitoring of lung pathologies, with higher accuracy compared to CXR [11]. Because of the anatomical advantages of a smaller thoracic cavity size, ribs that have not fully ossified, and a relatively thinner thoracic wall, the pediatric population may benefit greatly from LUS [12]. However, COVID‐19 pneumonia presents distinctive but not pathognomonic sonographic features; therefore, ultrasound is only to assess the possible lung involvement but cannot provide a specific etiological diagnosis of SARS‐CoV‐2 infection [13].

In COVID‐19 pneumonia, the main sonographic finding is bilateral diffuse interstitial syndrome, shown by B lines or vertical artifacts that appear patchy, along with irregularities of the pleural line, mostly in the posterobasal areas of the lungs. Consolidation may happen, indicating more advanced disease progression [14, 15]. These findings would correlate with tomographic findings considered typical of COVID‐19 including ground glass opacities, crazy‐paving pattern, and consolidations with bilateral and multifocal distribution, and a peripheral and posterior predominance [16]. It is important to note that the imaging finding of COVID‐19 pneumonia could be non‐specific and may be present in other pulmonary conditions, such as other viral pneumonias, connective tissue lung disease, and drug‐induced lung disease [17, 18]. Therefore, it is important to correlate the findings with clinical condition, temporal progression, and findings of other examinations [19].

Although pediatric patients are generally underrepresented in the literature compared to adults, several observational studies have examined the use of LUS in children since the emergence of COVID‐19 [20, 21, 22, 23, 24]. These studies not only explored sonographic features for monitoring and evaluating pneumonia during the acute phase but also highlighted the utility of LUS in detecting COVID‐19‐related complications such as multisystem inflammatory syndrome in children (MIS‐C) [25, 26, 27], pneumothorax [28], or acute respiratory distress syndrome [29]. More recent research has also reported on the role of LUS in post‐COVID follow‐up, particularly in assessing long‐term pulmonary effects [30, 31, 32].

There remains a need to further explore the clinical utility of LUS in the context of respiratory disease outbreaks, such as the recent COVID‐19 pandemic. This scoping review aims to build a comprehensive body of knowledge by synthesizing current evidence on the use of LUS in pediatric COVID‐19. Specifically, it will summarize existing findings, map the scope and nature of current research, and identify gaps to guide future investigations. Ultimately, by consolidating available literature, this review may support the strategic integration of LUS into pediatric care and enhance preparedness for future respiratory outbreaks.

### 1.1 Review Objective

To explore and synthesize current evidence regarding the role of LUS in pediatric COVID‐19, to summarize existing findings, map the scope and nature of current research, and identify gaps to guide future investigations.

## 2 Eligibility Criteria

### 2.1 Participants

Children of less than 18‐year‐old with clinical condition of suspected, confirmed, or recovered COVID‐19 infection.

### 2.2 Concept

The clinical use, diagnostic value, findings, and role of LUS in pediatric COVID‐19 cases. LUS examinations can be conducted at the point of care, by a clinician, as well as a traditional approach performed by a sonographer and interpreted by a clinician. We will consider all possible technical parameters related to the instrument and protocol, such as the ultrasound system and probe model, examination protocol, including lung zones or sides, scoring system, probe orientation, and body position. Studies using ultrasound solely for cardiac or vascular assessment will be excluded unless lung‐related sonographic features were also assessed and reported.

### 2.3 Context

All clinical healthcare settings during and after the COVID‐19 pandemic will be considered, including outpatient clinics, inpatient hospital wards, emergency departments, and intensive care units.

### 2.4 Types of Studies

Primary studies with experimental—including quasi‐experimental—and observational designs (e.g., cohort, case‐control, and cross‐sectional) will be considered for inclusion. This review will also consider the inclusion of descriptive reports, such as case series and individual case reports. Only review studies employing a systematic approach, including systematic reviews, with or without meta‐analysis, scoping reviews, and umbrella reviews, will be considered for inclusion.

Studies published from December 2019 will be included, as COVID‐19 was first emerging around that time. All relevant articles will be included regardless of language. Non‐English language reports will be screened using Google Translate (Alphabet Inc., CA, USA). If deemed relevant to the review, the articles will be translated with consultation by a native speaker.

## 3 Methods

This scoping review will be conducted in accordance with JBI methodological guidance for scoping reviews [33]. To ensure comprehensive and transparent reporting, the review will follow the preferred reporting items for systematic reviews and meta‐analyses extension for scoping reviews (PRISMA‐ScR) checklist [34]. In accordance with the guidelines, our scoping review protocol was registered in Open Science Framework (https://doi.org/10.17605/OSF.IO/XP628).

### 3.1 Search Strategy

The search strategy was developed in consultation with a medical librarian. The strategy aims to identify both published and unpublished studies. An initial limited search was conducted to locate relevant articles, review existing search strategies, and identify previously published work on the topic. The text words in the titles and abstracts of relevant articles and the index terms used to describe these articles were used to develop a complete search strategy for MEDLINE (Ovid) (see Table 1). This strategy, including all identified keywords and index terms, will be adapted for use in other databases. The following databases will be searched: MEDLINE (Ovid), Embase (Ovid), Web of Science, Scopus, and CINAHL (EBSCOhost).

**Table 1:** Search strategy on MEDLINE (Ovid), conducted at July 28, 2025.

No	Searches	Hits
1	exp ultrasonography/or ((ultrasound* or sonograph* or ultrason*) adj3 (lung or thoracic or chest or pulmona*)).ti,ab,kf.	516,720
2	exp Child/or exp Pediatrics/or exp Infant, Newborn/or exp adolescent/or (child* or p$ediatric* or adolescen* or infan* or neonat* or newborn).ti,ab,kf	499,0611
3	exp COVID‐19/or exp SARS‐CoV‐2/or (covid* or SARS$CoV$2 or coronavirus or corona).ti,ab,kf	496,005
4	1 and 2 and 3	296

Gray literature will be explored through searches in relevant databases such as Google Scholar, ProQuest Dissertations and Theses Global (PQDT), preprint platforms, and open‐access repositories (such as medRxiv, bioRxiv, SSRN, Zenodo). To further identify relevant materials, backward citation searching of reference lists and forward citation tracking of key articles will also be performed.

All searches will be re‐run 6 months prior to manuscript submission to ensure currency and completeness. The results of the search strategy, including the newly identified studies retrieved from re‐run, will be documented in the PRISMA flow diagram and a supplementary search appendix.

### 3.2 Study Selection

Following the search, all citations retrieved across databases will be imported and collated into the Covidence systematic review software (Veritas Health Innovation, Melbourne, Australia), and deduplication will be conducted. A pilot test of the study screening process will be conducted on a 10% sample of the total citations to ensure consistency and reliability. Two reviewers will independently assess the sample against the predefined inclusion criteria. Discrepancies will be discussed to evaluate interreviewer agreement, clarify ambiguities, and refine the inclusion criteria as necessary.

Following the pilot test, a two‐step process of screening against the predefined inclusion criteria will be conducted independently by two reviewers, that is, initial screening of titles and abstracts, followed by full‐text articles for review of eligible items. Any disagreements between reviewers at any stage will be resolved through discussion to reach a consensus.

Potentially relevant full‐text articles will be retrieved for detailed assessment. If full‐text articles are not readily available, efforts of retrieval will be made by contacting the authors up to two times. We will report results of the study selection process, including the reasons for exclusion of full‐text articles that do not meet the inclusion criteria, in the final scoping review and presented in a PRISMA flow diagram.

### 3.3 Data Extraction

Similarly, a pilot test will be conducted at the beginning of the data extraction phase. Two reviewers will independently extract data from 10% of the eligible full‐text articles using a draft data extraction tool (Table 2). The extracted data will be compared and discussed to resolve discrepancies and to ensure a consistent understanding of both the tool and the specific data items being collected. The draft tool—developed collaboratively by the reviewers—includes key elements such as study design, population characteristics, and a structured summary of key findings aligned with the review objectives. Once consensus and consistent application of the tool have been achieved through the pilot test, one reviewer will independently extract data from the remaining studies, while the second reviewer will verify the extracted data. As data extraction is an iterative process, the tool may be modified and refined throughout, as necessary. Any disagreements during this process will be resolved through discussion to reach a consensus.

**Table 2:** Data extraction table.

Items^a^	Sub‐item
Study information	author name; year; location; study design (e.g., prospective, retrospective, diagnostic accuracy)
Subject characteristics	number of subjects; gender; specific age range or group (e.g., neonate, toddler, adolescent); vaccination status
Examination comparator	molecular test, such as PCR; serial LUS; CXR; chest CT; other clinical or laboratory tests
Clinical condition	in relation to COVID‐19 (suspect, confirmed, post‐infection); level of severity (e.g., asymptomatic, mild, moderate, severe, critical); other condition (e.g., mechanically ventilated, MIS‐C, ARDS); comorbidity (e.g., asthma, immunocompromised, etc.)
Clinical purpose of LUS	specific applications of LUS in pediatric patients, including detection of lung involvement, assessment of disease severity, and acute complications—may include a report on typical sonographic features, monitoring progression, guiding management decisions (e.g., alveolar recruitment, physiotherapy, noninvasive ventilation, high‐flow nasal cannula), and follow‐up care, including assessment of long‐term complications.
Image acquisition and interpretation approach	protocol‐based, pattern‐based, or scoring systems, and composite of clinical or laboratory–ultrasound criteria.
Evidence‐generation methodology	expert consensus, feasibility, or validation studies on COVID‐related LUS application; the use of LUS against reference standards or other clinical or laboratory examinations, as in diagnostic test accuracy or concordance studies, reliability (inter and intrarater) studies, and operator training.
Study setting	general community; outpatient settings; hospital care (inpatient ward, emergency, or intensive care settings)
Technicalities	ultrasound system (brand, type of probe, and frequency); lung zones or sides; scoring system; probe orientation (i.e., parallel, perpendicular, or oblique); body position
Conclusion	key findings and study limitation

^a^ As applicable.

Studies that apply a different upper age threshold (e.g., 19 or 21 years) will be considered for inclusion if they explicitly define the population as pediatric or report data separately for individuals under 18 years of age. These studies will be assigned a specific code (e.g., “different age threshold”). For studies involving mixed‐age populations, inclusion will be considered only if data specific to individuals under 18 years are clearly extractable or can be obtained from the authors. In all such cases, efforts will be made to obtain pediatric‐specific data by contacting authors up to two times. If no response is received or if the relevant data are unavailable, the study will not be included in the synthesis but will be documented and assigned an exclusion code (e.g., “mixed‐age data not extractable”) to ensure transparency. These records will be presented in the PRISMA flow diagram and reported in a supplementary table. An additional supplementary table will list studies with missing or not extractable data, the number of authors contacted, response rates, number providing usable data, time to response, and how any author‐provided data contributed to the synthesis or analysis.

### 3.4 Synthesis of Results

Data synthesis will employ a deductive approach with inductive flexibility [35]. Included studies will be organized according to the period of data collection relative to the COVID‐19 pandemic (during COVID‐19 and post‐COVID‐19) to allow temporal comparison. Within each period, analysis will be guided by a set of predefined topics, while maintaining flexibility to capture additional themes emerging from the data.

The set of predefined topics will include,

- Clinical purpose of LUS. Including specific applications of LUS in pediatric patients, including detection of lung involvement, assessment of disease severity and acute complications, monitoring progression, guiding management decisions (e.g., alveolar recruitment, physiotherapy, noninvasive ventilation, high‐flow nasal cannula), and follow‐up care, including assessment of long‐term complications.
- Approach to image acquisition and interpretation. Including protocol‐based, pattern‐based, or scoring systems, and composite of clinical or laboratory–ultrasound criteria.
- Evidence‐generation methodologies. Including expert consensus, observational derivation, feasibility or validation studies on COVID‐related LUS application, studies on the use of LUS against reference standards or other clinical or laboratory examinations—as in diagnostic test accuracy or concordance studies, reliability (inter and intrarater) studies, and study as part of operator training.

Within each topics and period, studies will be analyzed to identify themes, emerging and recurring patterns. Inductive refinement will be applied if new concepts appear not captured a priori.

Relevant findings will be summarized, mapped, and presented in tables, with key contexts organized into data‐driven categories derived from the included studies. The results will be reported descriptively through a narrative summary to highlight the scope and nature of the available evidence and to identify gaps to guide future investigations. This approach will allow the review to clearly articulate both the breadth of existing pediatric LUS evidence and the evolution of its clinical applications, supporting identification of priority areas for future investigation.

## 4 Conclusion

There is a growing interest in the use of LUS in pediatric clinical practice, particularly during the COVID‐19 pandemic. Therefore, the authors have planned a scoping review to comprehensively examine the available literature on the use of LUS in pediatric COVID‐19. This review will explore temporal trends in LUS use during the pandemic, as well as extract and analyze the key topics highlighted in the literature. Ultimately, it aims to support the integration of LUS into clinical practice and to inform preparedness for future respiratory outbreaks.

## Author Contributions

**Yusuf Syaeful Nawawi:** conceptualization, methodology, writing – original draft, supervision. **Anistyaning Wahyu Adhie:** project administration, writing – review and editing, methodology. All authors have read and approved the final version of the manuscript.

## Conflicts of Interest

The authors declare no conflicts of interest.

## Transparency Statement

The lead author Yusuf Syaeful Nawawi affirms that this manuscript is an honest, accurate, and transparent account of the study being reported; that no important aspects of the study have been omitted; and that any discrepancies from the study as planned (and, if relevant, registered) have been explained.

## Data Availability

No datasets were generated or analyzed during this study. All materials supporting this protocol, including the data extraction form and supporting tables, are included within the manuscript.
